# RanBP2 regulates the anti-retroviral activity of TRIM5α by SUMOylation at a predicted phosphorylated SUMOylation motif

**DOI:** 10.1038/s42003-018-0198-0

**Published:** 2018-11-15

**Authors:** Ghizlane Maarifi, Juliette Fernandez, Débora M. Portilho, Aude Boulay, Jacques Dutrieux, Stéphane Oddos, Gillian Butler-Browne, Sébastien Nisole, Nathalie J. Arhel

**Affiliations:** 1 Institut de Recherche en Infectiologie de Montpellier (IRIM), Université de Montpellier, CNRS, 34090 Montpellier, France; 2 Saint-Louis Hospital, INSERM, Paris 7 University, 75010 Paris, France; 3 Bioaxial SAS, 75014 Paris, France; 4 Center for Research in Myology, INSERM, CNRS, Paris 6 University, 75013 Paris, France

## Abstract

TRIM5α is a cytoplasmic restriction factor that blocks post-entry retroviral infection. Evidence suggests that its antiviral activity can be regulated by SUMO, but how this is achieved remains unknown. Here, we show that TRIM5α forms a complex with RanGAP1, Ubc9, and RanBP2 at the nuclear pore, and that RanBP2 E3 SUMO ligase promotes the SUMOylation of endogenous TRIM5α in the cytoplasm. Loss of RanBP2 blocked SUMOylation of TRIM5α, altered its localization in primary cells, and suppressed the antiviral activity of both rhesus and human orthologs. In cells, human TRIM5α is modified on K84 within a predicted phosphorylated SUMOylation motif (pSUM) and not on K10 as found in vitro. Non-modified TRIM5α lacked antiviral activity, indicating that only SUMOylated TRIM5α acts as a restriction factor. This work illustrates the importance of the nuclear pore in intrinsic antiviral immunity, acting as a hub where virus, SUMO machinery, and restriction factors can meet.

## Abstract (web summary)

Ghizlane Maarifi et al. demonstrate that a nuclear pore component, RanBP2, SUMOylates the retroviral restriction factor TRIM5α to promote its antiviral activity. This study suggests an unexpected role of the nuclear pore for regulating anti-viral innate immunity.

## Introduction

Post-translational modifications by small ubiquitin-related modifiers (SUMO) can modulate protein stability, localization, and activity, to regulate numerous eukaryotic cellular functions such as gene expression, DNA repair, and chromatin organization^1,2^. In vertebrates, three SUMO paralogues can be covalently added onto substrate proteins: SUMO1, -2, and -3. SUMO2 and SUMO3 are almost identical in amino acid sequence and are therefore referred to as SUMO2/3. A SUMO4 variant exists but does not appear to be conjugated to cellular proteins^3^, and a SUMO5 paralogue has recently been described^4^. SUMO conjugation, known as SUMOylation, is brought about by the sequential action of three enzymes: an E1 SUMO-activating enzyme (SAE1/SAE2 heterodimer, Aos1/Uba2 in yeast), an E2 SUMO conjugating enzyme (Ubc9), and an E3 SUMO ligase such as protein inhibitor of activated STAT proteins (PIAS proteins)^5^, polycomb group protein Pc2^6^, and RanBP2^7^. The host SUMOylation system can play an important role in response to viral infection, both by activating cellular defenses that control viral replication and, conversely, serving as a target for viral evasion strategies that boost infection. Examples from the literature indicate that viral proteins can undergo SUMOylation or influence the SUMOylation of cellular proteins, and that the infection of some viruses is dependent on pathways that are regulated by SUMOylation^8^.

Restriction factors are antiviral proteins produced by the host to antagonise viral infection. They are considered the first line of defence against viruses, and can intervene literally within minutes of cell entry, to either degrade the virus or block its replication. A fine example is provided by the alpha isoform of the tripartite motif-containing protein 5 (TRIM5α), a cytoplasmic protein that prevents retroviruses from jumping the species barrier by intercepting viral capsids before they reach the nucleus. Although TRIM5α is constitutively expressed, experiments that modify the global cellular SUMO machinery profoundly affect TRIM5α-mediated restriction^9–11^, suggesting that the SUMO system provides a way for a host to modulate the antiviral activity of restriction factors. TRIM5α itself can be directly modified both by SUMO1 and SUMO2^11^, however no effect on its antiviral activity could so far be demonstrated.

Enzymes of the SUMO machinery are enriched in the nucleus and most SUMO substrates are nuclear proteins involved in nuclear processes such as transcription and DNA repair. Although TRIM5α forms cytoplasmic bodies, it can also shuttle to the nucleus^12,13^, suggesting that it could interact with SUMO machinery there. Some have shown co-localization of overexpressed TRIM5α with Promyelocytic Leukaemia Nuclear Bodies (PML-NB)^13,14^, which are a preferred site of SUMOylation and harbor many proteins of the SUMO machinery, including SUMO and PIAS1^15,16^, however we could not reproduce these findings with endogenous TRIM5α^13^. Therefore, although PIAS1 can SUMO modify TRIM5α in vitro^11^, their distinct endogenous localizations in cells suggests that PIAS1 is unlikely to act as the main E3 SUMO ligase of TRIM5α in vivo. In addition, treatment with ginkgolic acid, an inhibitor of the E1 SUMO-activating enzyme, reduces nuclear residency of TRIM5α^13^, suggesting that TRIM5α is SUMO modified in the cytoplasm and shuttles to the nucleus as a SUMOylated protein. Cytoplasmic SUMOylation has already been described for some proteins with restricted cytoplasmic localization, such as RanGAP1, indicating that enzymes of the SUMO machinery are also present in the cytoplasm^17^. We therefore set out to identify elements of the SUMO machinery that account for SUMO conjugation of TRIM5α in the cytoplasm.

Here, we show that TRIM5α associates with RanGAP1, RanBP2, and Ubc9 at the nuclear pore, and is SUMO conjugated by the nucleoporin and E3 SUMO ligase RanBP2 within this complex. In SUMO overexpressing cells, RanBP2 promoted both SUMO1 and SUMO2/3 conjugation on at least two sites. A 33 kDa C-terminal domain of RanBP2 previously shown to contain E3 ligase activity was sufficient to modify TRIM5α in an in vitro SUMO assay. RanBP2 dramatically impacted the localization of TRIM5α in two primary cell models and was required for efficient restriction of HIV-1 by rhesus TRIM5α in HeLa cells. This is the first report of RanBP2 participating in cellular intrinsic immunity mechanisms by regulating the antiviral activity of a restriction factor via its E3 SUMO ligase domain.

## Results

### TRIM5α binds to RanGAP1 and RanBP2 at the nuclear pore

**Fig. 1:**
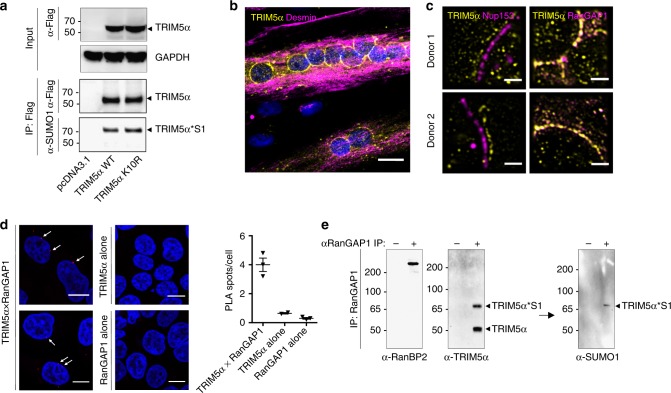
TRIM5α and SUMO-modified TRIM5α form a complex with RanGAP1 and RanBP2 at the nuclear pore. **a** The consensus site for in vitro SUMOylation by PIAS1 is not a target for SUMOylation in human cells. HEK 293T cells were transfected with Flag-tagged human TRIM5α or K10R mutant, previously shown to abrogate PIAS1-mediated SUMOylation in vitro. After 3 days, TRIM5α was immunoprecipitated using α-Flag antibodies and probed with α-SUMO1 or α-Flag antibodies. SUMO-modified TRIM5α is detected at ca. 70 kDa in the SUMO1 blots for both WT and K10R lanes (TRIM5α*S1). Western blots are representative of 3 independent experiments (original blots in Supplementary Figure 2a). **b** TRIM5α localizes at the nuclear envelope in myotubes. To identify the E3 SUMO ligase responsible for TRIM5α SUMOylation in cells, we searched for cell types in which endogenous TRIM5α did not form typical cytoplasmic aggregates, and probed for proximity with SUMO machinery. In human myotubes, the majority of TRIM5α signal accumulated around the nuclear envelope. Scale bar = 20 μm. **c** TRIM5α co-localizes with RanGAP1 in myotubes. Super-resolution imaging of human myotubes shows TRIM5α co-localization with Ran-specific GTPase activating protein RanGAP1, present on cytoplasmic filaments of the nuclear pore, but not with Nup153, which is found in the nuclear basket. Donor 1: neonatal myoblasts (CHQ), donor 2: adult myoblasts (160 M). Scale bar = 2 μm. **d** TRIM5α co-localizes with RanGAP1 in HeLa and HEK 293T cells. Proximity ligation assay was performed in HeLa and 293T cells, only HeLa are shown as an example. TRIM5α×RanGAP1 PLA signals were detected at a frequency of 4 and 1.5 spots/cell, respectively, and localized to the nuclear membrane in 55% of cases in both cells types, confirming that TRIM5α co-localizes with RanGAP1 at the nuclear envelope in these cells. Scale = 10 μm. **e** TRIM5α and SUMO-modified TRIM5α form a complex with RanGAP1 and RanBP2 in HeLa cells. RanGAP1 was immunoprecipitated from HeLa cytoplasmic extracts and precipitates were probed for TRIM5α and SUMO1. RanGAP1 immunoprecipitates contained RanBP2, unmodified TRIM5α (55 kDa), and a ~70 kDa TRIM5α species that also appeared in SUMO1 blots identifying it as SUMO1-modified TRIM5α (TRIM5α*S1). SUMOylated RanGAP1 (~90 kDa) is present at very low levels in cytoplasmic extracts and is therefore not detected here. Western blots are representative of 2 independent experiments (original blots in Supplementary Figure 2b)

Previous work showed that TRIM5α can undergo SUMO modification in vitro in the presence of recombinant SUMO, E1, E2, and E3 ligase PIAS1^11^. A TRIM5α mutant at the SUMOylation consensus site (K10R) is a poor substrate in this in vitro reaction, but maintains wild-type antiviral activity in cells, casting doubt on the relevance of PIAS1-mediated SUMOylation on TRIM5α function. To address this apparent discrepancy, we assessed the SUMOylation status of both wild-type and K10R mutant TRIM5α in cells. Flag-tagged TRIM5α was overexpressed and purified by immunoprecipitation from HEK 293T cell extracts. Blotting with an α-SUMO1 antibody revealed a ~70 kDa band, i.e., ~15 kDa above unmodified TRIM5α, in both wild-type and K10R transfected cells, indicating that the K10R mutant is SUMOylated in cells (Fig. 1a). These results, together with the fact that PIAS1 and TRIM5α are present in distinct subcellular compartments, indicates that PIAS1 cannot SUMOylate TRIM5α in vivo.

We reasoned that the E3 SUMO ligase responsible for TRIM5α SUMOylation in vivo is likely to be cytoplasmic since our previous work suggested that TRIM5α undergoes SUMOylation in the cytoplasm^13^. To identify elements of the SUMO machinery involved in the regulation of TRIM5α function, we searched for cell types in which endogenous TRIM5α did not form typical cytoplasmic aggregates, and investigated whether this altered localization correlated with a proximity to elements of the SUMO pathway. We identified an unusual pattern for TRIM5α in human myotubes, where the majority of the signal accumulated around the nuclear envelope (Fig. 1b). Since the nuclear pore is a privileged site for SUMOylating and deSUMOylating enzymes^18^, we asked whether TRIM5α co-localized with elements of the nuclear pore in these cells. Super-resolution imaging revealed a co-localization of TRIM5α with the Ran-specific GTPase activating protein RanGAP1, which is present on cytoplasmic filaments of the nuclear pore, but not with Nup153, which is found in the nuclear basket (Fig. 1c), indicating that TRIM5α is present at the cytoplasmic face of the nuclear pore in these cells.

An accumulation of TRIM5α around the nuclear membrane was not observed in other tested cell types, suggesting that this phenomenon might be transient in most systems. To determine whether TRIM5α can co-localize with RanGAP1 in other cells, we performed proximity ligation assay (PLA) in HeLa and HEK 293T cells. TRIM5α×RanGAP1 PLA signals were detected at a frequency of 4 and 1.5 spots/cell, respectively, and localized to the nuclear membrane in 55% of cases in both cells types, indicating that TRIM5α can co-localize with RanGAP1 at the nuclear pore in these cells (Fig. 1d).

To assess whether TRIM5α can interact with RanGAP1 in HeLa cells, we purified endogenous RanGAP1 by direct immunoprecipitation and blotted for possible interacting partners. RanGAP1 immunoprecipitated RanBP2, as expected since these have been shown to form a stable complex in cells^19^ (Fig. 1e). Endogenous TRIM5α was also found in RanGAP1 immunoprecipitates, both as the unmodified 55 kDa species, and a ~70 kDa protein that also appeared in SUMO1 blots identifying it as SUMO1-modified TRIM5α (TRIM5α*S1) (Fig. 1e). Results indicated that both unmodified and SUMO-modified TRIM5α are present in a complex with two major components of the nucleocytoplasmic transport machinery and key elements of the SUMO system, RanGAP1 and RanBP2.

### RanBP2 E3 SUMO ligase promotes SUMO1 conjugation of TRIM5α in cells

**Fig. 2:**
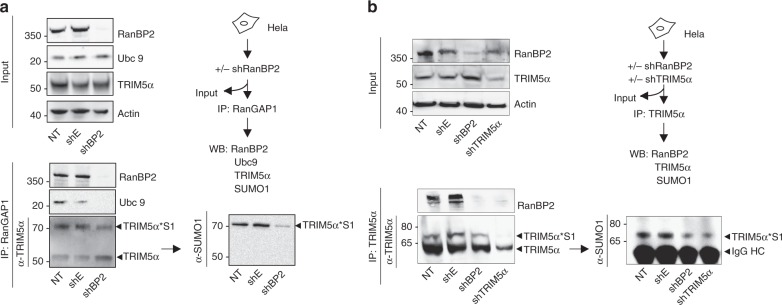
RanBP2 promotes TRIM5α SUMOylation within **a** TRIM5α/RanGAP1/RanBP2/Ubc9 complex. HeLa cells were transduced with a lentiviral vector coding for a shRNA against RanBP2 (shBP2), shTRIM5α, empty vector (shE), or not transduced (NT). After 3 days, cells were lysed and immunoprecipitated with **a** α-RanGAP1 or **b** α-TRIM5α antibodies. Input samples were analyzed by immunoblotting with a α-RanBP2, α-TRIM5α, α-Ubc9, and α-Actin antibodies, while immunoprecipitates were probed with α-Ubc9, α-RanBP2, α-TRIM5α, or α-SUMO1 antibodies. Higher molecular weight bands indicate SUMO-modified TRIM5α (TRIM5α*S1). Western blots are representative of 2 and 3 independent experiments, respectively (original blots in Supplementary Figures 3)

To address whether RanBP2 can promote TRIM5α SUMO modification within the RanGAP1/RanBP2 complex, we isolated endogenous TRIM5α by RanGAP1 immunoprecipitation from cells expressing or not RanBP2. Transduction of HeLa cells with a specific RanBP2 shRNA^20^ resulted in efficient knockdown of RanBP2 (Fig. 2a, input). RanGAP1 immunoprecipitates from non-transduced and control vector transduced cells contained RanBP2, Ubc9, TRIM5α, and TRIM5α*S1, indicating that these are present together in a complex in cells. As previously, TRIM5α*S1 was identified as a ~70 kDa band that was visible in both TRIM5α and SUMO1 blots. Both Ubc9 and RanBP2 were absent from RanGAP1 immunoprecipitates in RanBP2 depleted cells, indicating that Ubc9's association with the RanGAP1/RanBP2 complex is dependent upon its interaction with RanBP2. This was not found to be the case for TRIM5α, which was still detected in RanGAP1 immunoprecipitates from RanBP2 knockdown cells, suggesting that its association with RanGAP1 can be maintained in the absence of RanBP2. Critically, TRIM5α*S1 levels were reduced and unmodified TRIM5α levels were increased in the absence of RanBP2, indicating that RanBP2 promotes SUMO1 modification of TRIM5α in cells (Fig. 2a).

To confirm that TRIM5α is a target for SUMO1 modification by RanBP2 E3 SUMO ligase, we performed the reverse experiment and immunoprecipitated endogenous TRIM5α directly from HeLa cells treated with shRNAs against RanBP2 or TRIM5α (Fig. 2b). RanBP2 was present in TRIM5α immunoprecipitates, and disappeared as expected in the absence of either RanBP2 or TRIM5α, confirming that TRIM5α is present in a complex with RanBP2 in cells. As previously, a TRIM5α*S1 band was detected in both TRIM5α and SUMO1 blots, and decreased in cells depleted of RanBP2. The same band also decreased in cells treated with a specific TRIM5α shRNA, confirming its specificity. Together, results confirmed that RanBP2 promotes TRIM5α SUMOylation in cells.

### RanBP2 promotes multiple conjugation of SUMO1 and SUMO2/3 on TRIM5α

**Fig. 3:**
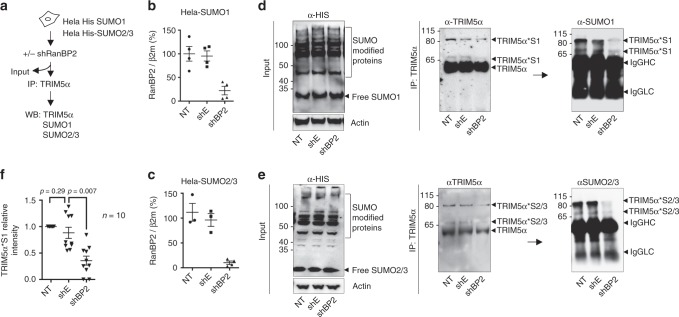
RanBP2 promotes multiple conjugation of both SUMO1 and SUMO2/3 on TRIM5α. **a** Schematic representation of the experimental procedure. **b** HeLa-SUMO1, and **c** HeLa-SUMO2/3 cells were transduced with shBP2, shE, or not transduced (NT). After 3 days, RanBP2 depletion was assessed by qPCR. TRIM5α was immunoprecipitated from **d** HeLa-SUMO1 and **e** HeLa-SUMO2/3 cells. Input samples were analyzed by immunoblotting with a α-His antibody to reveal SUMO-modified proteins, while TRIM5α immunoprecipitates were probed with α-SUMO1 or α-SUMO2/3 antibodies. Higher molecular weight bands indicate SUMO-modified TRIM5α (TRIM5α*S1 and TRIM5α*S2/3). IgG heavy (HC) and light chains (LC) are indicated. Western blots are representative of 3 independent experiments (original blots in Supplementary Figure 4). **f** TRIM5α*S1 bands were quantified from 10 independent SUMO blots of TRIM5α immunoprecipitates. The graph shows the mean relative intensity of SUMO bands normalized for NT controls ± SEM. Statistical analysis was performed using a paired *t* test

Previous work suggested that TRIM5α can undergo conjugation to SUMO1 in vitro on up to three distinct lysines^11^. Since our experiments so far only allowed detection of a single SUMO-modified TRIM5α band, we tested whether overexpression of SUMO in cells might promote detection of other higher molecular bands. We therefore repeated TRIM5α immunoprecipitation from His-SUMO1 and His-SUMO2/3 overexpressing HeLa cells, depleted or not for RanBP2 for 3 days (Fig. 3a–c). SUMO expression in cells was probed using α-His antibody on input samples (Fig. 3d, e). Endogenous TRIM5α was then immunoprecipitated and blotted using an α-TRIM5α antibody to control for successful immunoprecipitation, and α-SUMO1 or α-SUMO2/3 antibodies to reveal TRIM5α SUMOylation (Fig. 3d, e, respectively). In SUMO1 and SUMO2/3-expressing cells, two high molecular weight bands of ~70 and ~80 kDa were observed in control non-transduced and shEmpty samples, species that have already been described for TRIM5α^11^ (Fig. 3d, e). The ~80 kDa band likely corresponds to the attachment of single SUMO1 entities at two separate lysines in TRIM5α, although we cannot exclude that it could also reflect the linkage of two SUMO1 moieties on the same lysine^7^. TRIM5α*S1 and TRIM5α*S2/3 bands appeared in both TRIM5α and SUMO blots, but were reduced in cells depleted for RanBP2 (Fig. 3f), indicating that RanBP2 can promote both SUMO1 and SUMO2/3 modification on at least two sites on TRIM5α in HeLa cells.

### The E3 SUMO ligase domain of RanBP2 modifies TRIM5α in vitro

The E3 SUMO ligase activity was previously shown to be contained within a 33 kDa C-terminal domain of RanBP2 (BP2∆FG)^7^. To determine whether BP2∆FG is sufficient to SUMOylate TRIM5α, we set up an in vitro SUMOylation assay for human recombinant His-tagged TRIM5α (~40 kDa), with recombinant SUMO1, E1, E2, and the small E3 fragment BP2∆FG. Increasing amounts of BP2∆FG were added to an otherwise constant reaction mix and control reactions were carried out in reaction mix lacking ATP, E2, or E3. Samples were analyzed by immunoblotting with α-SUMO1 and α-His antibodies. As expected, no SUMO1 modification could be detected in the absence of either ATP, E2, or BP2∆FG (Supplementary Figure 1a). However, adding BP2∆FG to the reaction stimulated the appearance of TRIM5α*S1 bands at roughly 15 kDa increments above the 40 kDa unmodified recombinant TRIM5α band: ~55, ~70, ~85 kDa, and so forth (Supplementary Figure 1a). This effect was dose-dependent for the first ~55 kDa band, but was inverse dose-dependent for higher molecular weight bands, suggesting that excess BP2∆FG competes with TRIM5α for SUMO1. Results demonstrate that RanBP2 is sufficient to SUMO-modify TRIM5α, and that SUMOylating activity is contained within the small BP2∆FG domain. Full-length HeLa RanBP2 was also found to stimulate TRIM5α SUMOylation in in vitro reactions (Supplementary Figure 1b), however, we cannot exclude that modified TRIM5α is carried over by RanBP2 precipitated from HeLa cells.

### RanBP2 is required for rhesus TRIM5α-mediated restriction of HIV-1

**Fig. 4:**
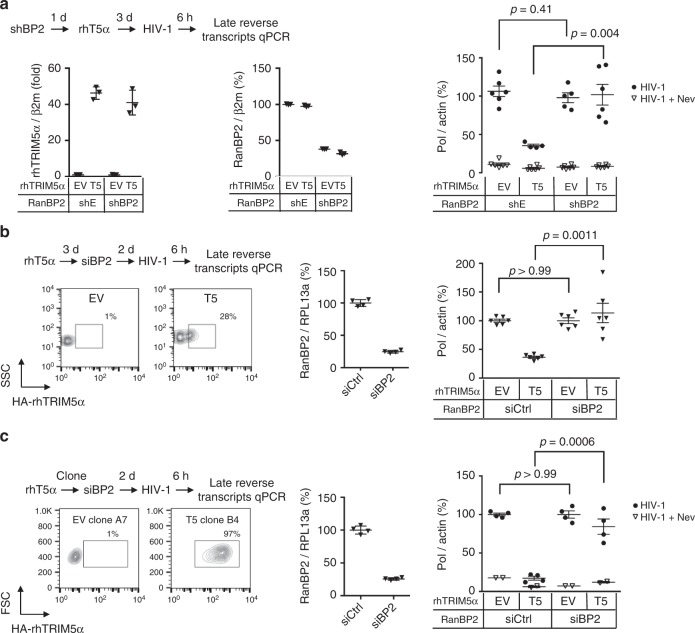
RanBP2 regulates the anti-HIV-1 activity of rhesus TRIM5α. **a** HeLa cells were transduced with shBP2 or shE at d0, transduced with rhesus TRIM5α (T5) or empty vector (EV) at d1, and infected with VSV-G pseudotyped HIV-1 at MOI 1 at d5 in the absence or presence of the reverse transcription inhibitor Nevirapine (Nev). RanBP2 and TRIM5α transcripts were quantified by qRT-PCR and normalized for β2-microglobulin. To determine the efficiency of reverse transcription, Pol DNA transcripts were quantified at 6 hpi by qPCR and normalized for actin. Results show the mean of 3 experiments performed with independent lentiviral vector stocks ± SD. Unpaired *t* test was performed on HIV-1 data sets using Prism 6. Expression of rhTRIM5α led to a decrease in Pol copy numbers in control samples following HIV-1 infection, indicating efficient TRIM5α-mediated restriction. Depletion of RanBP2 cancelled the ability of TRIM5α to block HIV-1 reverse transcription, indicating that RanBP2 is key in mediating efficient TRIM5α restriction. **b** The same experiment was repeated but by first overexpressing rhTRIM5α then depleting RanBP2. TRIM5α expression was monitored by flow cytometry using HA labelling. Graphs are representative of two independent experiments. **c** To obtain a greater restriction phenotype, the experiment was repeated using rhTRIM5α cell clones. Graphs are representative of two independent experiments

TRIM5α is a restriction factor that antagonises infection of rhesus macaque cells by HIV-1^21^. Restriction operates by direct recognition of the viral capsid and blocks productive infection by arresting reverse transcription, required for the conversion of the viral RNA genome into double stranded DNA. Previous work showed that overexpression of SUMO1 in cells promoted TRIM5α antiviral function, although this was assumed to involve the interaction of unmodified TRIM5α with SUMOylated proteins via its SIM domains^9,10^. To determine whether SUMO modification of TRIM5α by RanBP2 can potentiate its antiviral function, we transfected rhesus TRIM5α (rhTRIM5α) in HeLa cells treated or not with a specific shRNA against RanBP2. Cells were then infected with HIV-1 and reverse transcription efficiency was assessed by quantitative PCR amplification of Pol DNA sequences at 6 h post-infection (hpi). Of note, we did not perform reporter assays since RanBP2 knockdown blocks HIV-1 nuclear entry^20^. Therefore the effect of RanBP2 on rhTRIM5 anti-HIV activity may only be reliably measured before the nuclear import step. In our assay, expression of rhTRIM5α led to a decrease in Pol copy numbers in control samples, indicating TRIM5α-mediated restriction (Fig. 4a). Transduction with a shRNA targeting RanBP2 did not affect reverse transcription in control cells, as shown previously^20^. Strikingly, however, depletion of RanBP2 cancelled the ability of TRIM5α to block HIV-1 reverse transcription. Similar results were obtained if rhTRIM5α was transfected prior to RanBP2 knockdown, and at all tested multiplicities of infection (MOI) (Fig. 4b).

To obtain a greater restriction phenotype, we generated HeLa clones overexpressing rhTRIM5α. In these cells, HIV-1 reverse transcription reached background levels similarly to those obtained with Nevirapine. Remarkably, the simple lipofection of an siRNA against RanBP2 in these stable clones, compared to control siRNA, blocked the ability of rhTRIM5α to restrict HIV-1 indicating that RanBP2 is key in mediating efficient rhTRIM5α restriction (Fig. 4c).

### RanBP2 modulates the antiviral activity of endogenous TRIM5α

**Fig. 5:**
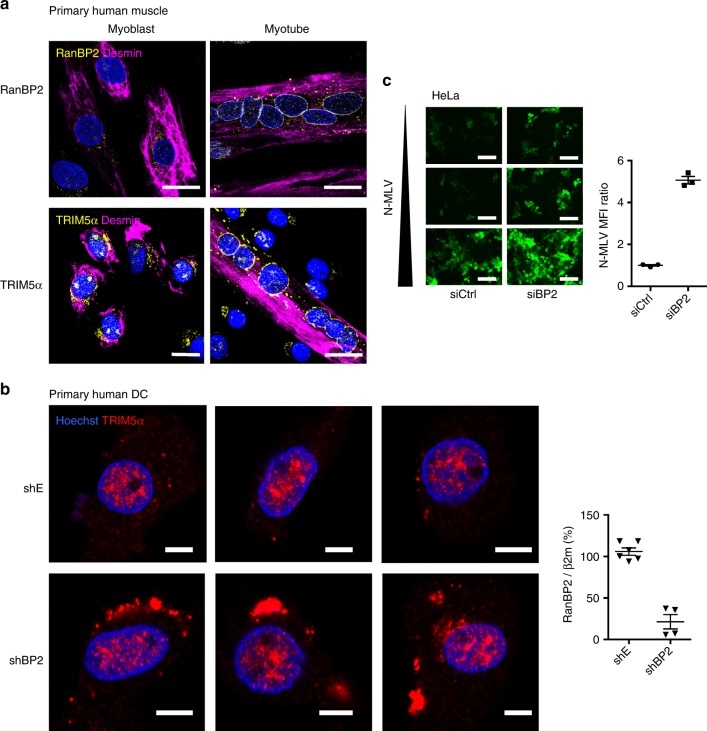
RanBP2 regulates the localization and antiviral restriction activity of human endogenous TRIM5α. **a** RanBP2 regulates the subcellular localization of TRIM5α in human myotubes. The localization of RanBP2 (top), and TRIM5α (bottom) were assessed in myoblasts and myotubes using confocal imaging. Scale bar = 20 μm. **b** RanBP2 regulates the subcellular localization of TRIM5α in human dendritic cells. Human monocyte-derived DCs were transduced with a RanBP2 shRNA (shBP2) or empty vector (shE). Knock-down was assessed at d7 by qRT-PCR and DCs were labelled with an α-TRIM5α antibody. Three representative fields are shown for each condition. Scale bar = 5 μm. **c** RanBP2 regulates the antiviral activity of endogenous TRIM5α. HeLa cells, which naturally restrict N-MLV, were lipofected with an siRNA against RanBP2 or control (siBP2 and siCtrl, respectively) and after 2 days were infected with increasing doses of N-MLV-YFP (MOI 1, 5, 10). Images were acquired at 2 dpt by widefield microscopy. The graph shows the mean fluorescence intensity (MFI) in representative fields at MOI 1. Scale bar = 100 μm

We previously showed that SUMOylation of TRIM5α can regulate its subcellular localization in specific cell types^13^. To determine whether SUMOylation of TRIM5α by RanBP2 can impact its subcellular localization, we returned to our muscle cell model in which TRIM5α localizes at the nuclear envelope (Fig. 1b). Previous work showed that skeletal muscle differentiation is associated with changes in nuclear pore architecture and composition^22^, including a dramatic up-regulation of RanBP2 protein expression and residence in nuclear pore complexes (NPC)^23^. We obtained primary myoblasts in which RanBP2 was barely detectable, and differentiated them into myotubes to induce strong expression of RanBP2 at the nuclear envelope. Interestingly, the absence of RanBP2 in the myogenic precursor cells was associated with the accumulation of TRIM5α in the cytoplasm away from the nuclear envelope (Fig. 5a). Although a direct correlation cannot be proven given that the differentiation of myoblasts to myotubes changes the expression of many genes^24^, these results suggest that induction of RanBP2 expression alters TRIM5α localization.

As a parallel example, we tested primary monocyte-derived DCs, in which we described a SUMO-dependent nuclear sequestration of TRIM5α^13^. Primary DCs were transduced with either empty or shRanBP2 lentiviral vectors and monitored for TRIM5α localization by confocal microscopy (Fig. 5b). In control cells, TRIM5α was almost exclusively nuclear, as previously reported^13^. In contrast, loss of RanBP2 led to a dramatic relocation of some TRIM5α signal to the cytoplasm as bright aggregates, suggesting that SUMOylation of TRIM5α by RanBP2 contributes to its nuclear targeting in DCs.

Having shown that RanBP2 mediates the antiviral activity of overexpressed rhTRIM5α, we examined whether it also affects the ability of N-MLV infection to be restricted by endogenous huTRIM5α. We lipofected human cells with an siRNA against RanBP2 or control, and infected with increasing doses of N-MLV-YFP. As expected, human TRIM5α restricts N-MLV at low doses and is saturated with increasing MOI. However, the depletion of RanBP2 led to a measurable increase in N-MLV infection efficiency at all tested viral inoculums, with a 5-fold increase in fluorescence intensity at MOI 1, indicating that RanBP2 regulates the antiviral activity of endogenous human TRIM5α (Fig. 5c).

### TRIM5α is SUMOylated on a pSUM consensus motif at position K84

**Fig. 6:**
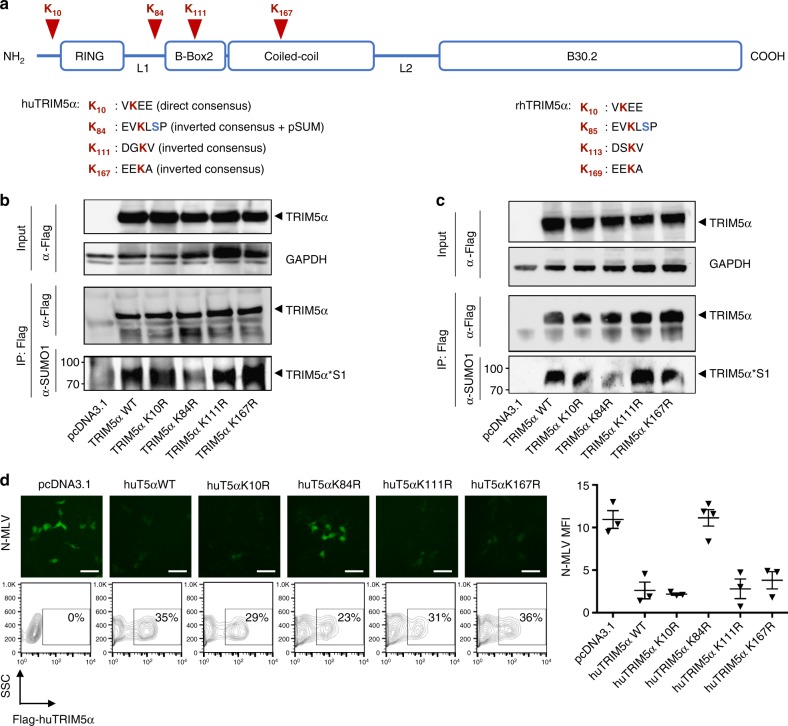
TRIM5α is SUMOylated on residue K84 within a predicted pSUM consensus motif. **a** Schematic representation of the human TRIM5α protein domains (to scale). The 4 lysines within putative consensus SUMO modification sequences are indicated in red. The corresponding sequences in rhesus TRIM5α are indicated on the right for comparison purposes. **b** HEK 293T (original blots in Supplementary Figure 5a) and **c** MDTF cells (original blots in Supplementary Figure 5b) were transfected with the indicated pcDNA human TRIM5α expression constructs. Inputs were monitored by Western blotting using anti-Flag and -GAPDH antibodies. TRIM5α was immunoprecipitated using anti-Flag beads as in Fig. 1a. TRIM5α SUMOylation was assessed by Western blotting on immunoprecipitated TRIM5α using anti-Flag and -SUMO1 antibodies. The TRIM5α*S1 band was readily detectable for all TRIM5α mutants but was noticeably reduced for the K84R mutant, indicating that SUMO is conjugated at this residue. Western blots are representative of 2 independent experiments. **d** SUMOylation on K84 is required for the antiviral function of TRIM5α. MDTF cells were transfected with the wild-type and mutant human TRIM5α constructs then infected with N-MLV at MOI 2. Top panels show representative fields of wide-field microscopy. Equal transfection efficiency was monitored by flow cytometry (plots below). Mean fluorescence intensity (MFI) was quantified by Fiji software. The graph shows the values from 3 independent fields ± SD and is representative of 2 independent experiments. Human TRIM5α and the K10R, K111R, and K167R mutants all restricted N-MLV, whereas the K84R mutant lost the ability to do so. Scale bar = 50 μm

Having established that the human TRIM5α K10R mutant is SUMOylated to wild-type levels in cells, we sought to identify the site of SUMO modification on TRIM5α. We generated 4 lysine mutants in strong direct and inverted SUMO consensus motifs as predicted by the JASSA bioinformatic tool^25^ (Fig. 6a) and tested them for their ability to be SUMOylated. In two different cell types, the K10R, K111R, and K167R mutants were all SUMOylated as wild-type TRIM5α. In contrast, the K84R mutant showed significantly reduced SUMOylation, indicating that this residue is the preferred site for SUMO modification in cells (Fig. 6b, c). This residue is located in the linker 1 region that connects the RING and B-box2 domains (Fig. 6a). Remarkably, K84 resides in a pSUM phosphorylated SUMOylation motif (yKXpSP)^25,26^ in addition to an antisense SUMOylation consensus motif^27^, similarly to the corresponding K85 residue in rhesus TRIM5α. Experimental evidence^28^ and prediction algorithms (PhosphoSite Plus) both confirm that S86 on human TRIM5α and S87 on rhesus TRIM5α are phosphorylated, suggesting that TRIM5α is SUMOylated on a consensus pSUM motif next to a phosphorylated serine.

We next tested the K84R mutant for its antiviral activity. Non-restrictive MDTF cells were transfected with the different human TRIM5α constructs. Transfection efficiency was found to be around 30% for all samples (Fig. 6d). The K10R, K111R, and K167R mutants all restricted N-MLV efficiently. In contrast however, the K84R mutant cancelled the antiviral activity of TRIM5α, indicating that SUMOylation at K84 is essential for TRIM5α function (Fig. 6d).

## Discussion

Restriction factors are considered the first line of defense against viruses, however little is known about how their antiviral function is regulated in cells. Evidence suggests that the antiviral activity of TRIM5α can be regulated by the SUMO machinery^9–11,13^ but how this is achieved was until now unknown. To our knowledge, we show for the first time that a nuclear pore component, Nup358 also called RanBP2, SUMOylates the retroviral restriction factor TRIM5α and promotes its antiviral activity.

RanBP2 was previously shown to promote SUMO conjugation of Sp100^7^, HDAC4^29^, and Mdm2^30^. This study identifies TRIM5α as a new substrate for RanBP2 E3 SUMO ligase. RanBP2 promoted single conjugation of SUMO1 moieties onto TRIM5α in HeLa cells, as well as multiple SUMO1 and SUMO2/3 conjugation in SUMO-overexpressing HeLa cells. Although the lysine at position 10 of TRIM5α is in a strong SUMOylation consensus motif, it is unlikely that RanBP2 mediates SUMOylation at this position, since K10R mutants are still SUMOylated in HeLa cells, and are unaffected in their restriction activity^11^. Indeed, we show that the lysine at position 84, which is in a predicted pSUM consensus motif and an antisense SUMO conjugation pattern, is the preferred site for SUMO1 modification in cells. Interestingly, the adjacent serine within the pSUM domain, S86 in human TRIM5α (PhosphoSite Plus algorithm) and S87 in rhesus TRIM5α^28^, is a major site for phosphorylation, suggesting that SUMO modification of TRIM5α at K84 is phosphorylation-dependent.

The primary effect of SUMO modification of TRIM5α by RanBP2 is likely to be to turn on TRIM5α's activity as an anti-retroviral factor. Depletion of RanBP2 abolished restriction of HIV-1 in rhesus TRIM5α overexpressing cells and blocked restriction of N-MLV by human endogenous TRIM5α. Moreover, the K84R mutant lost its ability to restrict incoming retroviruses, indicating that TRIM5α must be SUMOylated to carry antiviral activity. The presence of K84 within a phosphorylation-dependent SUMOylation pSUM domain implies that the activity of TRIM5α is turned on sequentially first by phosphorylation then SUMOylation. Further work is needed to determine whether mutating the phosphoserine within the pSUM blocks SUMOylation, and how TRIM5α phosphorylation is regulated. Intriguingly, only SUMOylated TRIM5α can antagonise incoming retroviruses, suggesting that a constitutively active restriction might be disadvantageous to the cell. Our results point to an elaborate intrinsic immunity mechanism that is mounted by the cell to regulate the antiviral function of a key restriction factor, suggesting that a constitutively active restriction might be disadvantageous to the cell.

In addition, RanBP2 modulates the subcellular localization of TRIM5α. In dendritic cells, where efficient sensing of invading pathogens is essential, SUMO modification of TRIM5α leads to its relocation to the nucleus where it is no longer able to neutralize incoming cytoplasmic viruses^13^. The absence of RanBP2 in these cells leads to a partial relocation of TRIM5α to the cytoplasm where it forms aggregates. Given this, it is likely that SUMOylation of TRIM5α by RanBP2 can both promote and inhibit (via nuclear sequestration) its activity as a retroviral restriction factor, and that this is fine-tuned according to the cell type (lymphocytes versus DC^13^) and whether the cell is undergoing infection. Similarly, other restriction factors such as PML/TRIM19 and Mx proteins have been shown to be regulated by SUMO, either by direct conjugation or via SIM domains^31^, underlining the importance of the SUMO machinery in intrinsic antiviral defense mechanisms.

SUMOylation is a highly transient post-translational modification, which likely explains why TRIM5α SUMOylated bands were more apparent when TRIM5α was enriched, either by RanGAP1 co-immunoprecipitation or direct TRIM5α immunoprecipitation, and in SUMO-overexpressing HeLa cells. Similarly, TRIM5α localization at the nuclear pore was rarely observed except by TRIM5α×RanGAP1 PLA, suggesting that its residency at the nuclear pore is a transient phenomenon. A striking exception was provided by myotubes, in which TRIM5α forms a perinuclear ring, which may be accounted for by the fact that RanBP2 is strongly induced during myogenic differentiation. The transient nature of SUMO modification of TRIM5α in all other systems is likely to be important to fine-tune its function and localization.

Besides direct conjugation of TRIM5α by SUMO variants^11^, which we confirm in this study, the SUMO machinery is likely to contribute in additional ways to TRIM5α function. Several SUMO-interacting motifs (SIM) identified in TRIM5α^9,10,14^ may mediate interaction with SUMOylated proteins. For instance, the physical association between TRIM5α and RanGAP1 that we describe in this study likely involves an interaction between SUMOylated RanGAP1 and SIM domains on TRIM5α, and may be key to bring TRIM5α in proximity of its E3 SUMO ligase, RanBP2.

Viruses that replicate in the nucleus, such as lentiviruses, exploit NPCs and their associated proteins for nuclear entry. However, the NPC is also a privileged site for SUMOylating and deSUMOylating enzymes^18^, indicating that the nuclear pore is likely to both facilitate and impair viral nuclear import, acting both as gate and gatekeeper of viral passage into the nucleus^32^. In the case of HIV, the nucleoporin RanBP2 promotes capsid docking at the nuclear envelope and infection^20,33,34^, but can also potentiate the antiviral functions of the restriction factor TRIM5α by mediating its SUMOylation.

Interestingly, some HIV proteins have been shown to interact with the SUMO machinery and undergo SUMOylation^35–37^, and further work will determine whether RanBP2 or more generally the SUMO machinery present at the NPC are involved in SUMOylating viral proteins during their transit into the nucleus.

Equally interesting is the presence of TRIM5α with RanGAP1/RanBP2/Ubc9 at the nuclear pore. Although TRIM5α is traditionally thought to intercept viral capsids during their rapid cytoplasmic trafficking towards the nucleus, the association of TRIM5α with cytoplasmic bodies is not required for its antiretroviral activity^38^. Given our findings, it is possible that TRIM5α can intercept capsids at the nuclear pore, where they accumulate for several hours prior to nuclear entry^39,40^, a bottleneck that constitutes a point of vulnerability in HIV infections. The possible presence of TRIM5α at the NPCs is reminiscent of MxB, another restriction factor that is also reported to operate at the cytoplasmic face of NPCs^41,42^.

Results obtained here and elsewhere point to an essential role for the nuclear pore and more specifically RanBP2 in lentiviral infections, acting as a hub where virus, SUMO machinery, and restriction factors can meet.

## Methods

### Cells

HeLa-SUMO1 and -3 cell lines were obtained by transducing HeLa cells (ATCC) with Puro-His-SUMO1, or -3 lentiviral vector. HEK 293T cells were obtained from the ATCC. *Mus dunni* tail fibroblast (MDTF) cells devoid of TRIM5α, APOBEC3G, and tetherin restriction factors, were used to titer the N-MLV vector^43^. Myoblasts were isolated from the quadriceps of donor 1 CHQ and donor 2 160M (aged 5 days and 53 years, respectively^44^), and differentiated into myotubes in low-serum DMEM 2% FBS for 5 days. Myotubes appear as polynucleated desmin-positive cells. Blood was obtained from healthy donors who signed an informed consent that their blood could be used for research purposes (Etablissement de Sang Français (EFS)). Dendritic cells were differentiated from blood monocytes as previously described^13^. The rhTRIM5α cell clones A7 and B4 (for EV and T5, respectively) were obtained by transducing HeLa cells with the empty LPCX or LPCX-HA-rhTRIM5α retroviral expression vectors^21^, selecting for puromycin resistance and performing limiting dilutions of the parent population. The clones were screened by flow cytometry (Novocyte, Ozyme) for HA expression.

### Antibodies

Primary antibodies used were mouse anti-6×His (Clontech), -desmin (Dako), -Nup153 (gift from B. Burke), -3xFlag (Sigma), -actin (Sigma), -GAPDH (Sigma), -Ubc9 (Abgent) and α-tubulin (Sigma); rabbit anti-RanBP2 (Pierce), -TRIM5α (gift from P. Bieniasz)^13,45^, -SUMO1 and -SUMO3 (Cell Signaling); goat anti-RanGAP1 (Santa Cruz Biotechnology); rat anti-HA (3F10, Roche). Secondary antibodies used were anti-mouse, -rabbit, -goat, -rat HRP (GE Healthcare), protein G-HRP used to avoid IgG detection when revealing immunoprecipitated TRIM5α, and Alexa-488, -555, and -647 (Invitrogen).

### RNA interference and quantitative PCR

Lentiviral vectors shE, shBP2, and shTRIM5α have been described previously^13,20^. The following siRNAs were used: siCtrl GAGAAGGUAAAGCUGGACAUU and siBP2 CACAGACAAAGCCGUUGAAUU. Hela, Hela-SUMO1, and Hela-SUMO3 cells (0.5 × 10^6^) seeded at d0 were transduced at d1 with MOI 50 lentiviral vector. Alternatively, cells were lipofected with 50 nM siRNA using RNAiMAX transfection reagent (Invitrogen). Realtime quantitative PCR (qPCR) reactions were performed on a QuantStudio 5 (Thermo Fisher Scientific) using the following primers and probes.

RBP2-F: ACAATGGAATTAAAGCCCTTAAATGT,

RBP2-R: GAAACAATCAGCTACTTCTTTAGTTTTA,

RBP2-P: FAM-TTGGACTGCCTCAGATTATGCTGATGGAGAAGCAA-3′BHQ1,

ß2M-F: GTGCTCGCGCTACTCTCTCT,

ß2M-R: CTCTGCTGGATGACGTGAGT,

ß2M-P: HEX-CGCTGGATAGCCTCCAGGCC-BHQ1,

Pol-F: TTTAGATGGAATAGATAAGGCCCAA,

Pol-R: CAGCTGGCTAACTATTTCTTTTGCTA,

Pol-P: FAM-AATCACTAGCCATTGCTCTCCAATTAC-TAMRA,

Actin-F: AACACCCCAGCCATGTACGT,

Actin-R: CGGTGAGGATCTTCATGAGGTAGT,

Actin-P: FAM-CCAGCCAGGTCCAGACGCAGGA-BHQ,

TRIM5-F: TTGGATCCTGGGGGTATGTGCTGG,

TRIM5-R: TGATATTGAAGAATGAGACAGTGCAAG,

RPL13A-F: CCTGGAGGAGAAGAGGAAAGAGA,

RPL13A-R: TTGAGGACCTCTGTGTATTTGTCAA.

### Viruses

HIV-1 was produced by transient co-transfection of 293T cells by calcium phosphate precipitation with the envelope defective proviral plasmid (pLAI-∆env) and the VSV-G envelope expression plasmid. Virus supernatants were titered by p24 ELISA assay (Clontech). The N-MLV vector was produced by co-transfection of pCFG2-YFP, pCIG3N, and pVSV-G. Vector supernatants were collected at 2 dpt, cleaned up through 0.45 µm filter, and concentrated by ultracentrifugation for 1 h at 22,000 rpm using a SW32Ti Beckman rotor. Vector stocks were titered in MDTF cells by flow cytometry.

### Fluorescence imaging

Widefield microscopy was performed using an Evos microscope (Life Technologies). Confocal microscopy and PLA were performed on a LSM880 microscope as previously described^11,13^. Quantification of PLA spots per cells was performed using Imaris v 9.0 (ImarisXT, Bitplane) on 4 different z-stacks (~200 nuclei) for each condition ± SD. Proximity of spots to the nuclear envelope was assessed using a nuclear cache within a 1 µm distance of the nuclear envelope. Super-resolution (80 nm) imaging was performed on a CoDiM 100 system (Bioaxial France) equipped with 488, 561, and 640 lasers (Toptica, Germany). The CoDIM system was mounted on a Nikon Eclipse upright confocal microscope (Nikon, Japan) equipped with 1.2NA water immersion and 1.49NA oil immersion objectives (Nikon, Japan). CoDim super-resolution imaging uses a biaxial crystal to project a variety of high-contrast Airy-disc sized excitation patterns with high spatial-frequency content on the sample. Resulting fluorescence images are then recorded on a low-noise Hamamatsu Orca flash 4 sCMOS camera (Hamamatsu, Japan) and final images are obtained using a proprietary maximum likelihood a posteriori image reconstruction algorithm as described in ref. ^46^.

### Site-directed mutagenesis

The K84R, K111R, and K167R human TRIM5α mutants were generated by site-directed mutagenesis using the QuikChange II site-directed mutagenesis kit (Agilent).

### Co-immunoprecipitation experiments

RanGAP1 and TRIM5α co-immunoprecipitation were carried out at 4 days post-transduction with lentiviral vectors shE or shBP2 using cytosolic extracts (prepared using NE-PER kit (Pierce)) and whole cell lysates, respectively, in the presence of 20 mM NEM (N-Ethylmaleimide, deSUMOylase inhibitor) and EDTA-free protease inhibitor cocktail (Roche). Supernatants were pre-cleared by incubation with 40 µl Novex recombinant protein G sepharose and 3 µl normal goat serum (Invitrogen) for 90 min at 4 °C followed by centrifugation at 350*g* for 5 min. Supernatants were then incubated with 100 µl goat α-RanGAP1 or 10 µl rabbit α-TRIM5α antibody and 40 µl protein G sepharose overnight at 4 °C. Beads were washed 5 times in lysis buffer before elution.

To detect SUMOylation of WT and mutant human TRIM5α constructs, HEK 293T or MDTF cells (2–3 × 10^6^) seeded at d0 were transfected with 10 µg of pcDNA3.1 empty vector, or Flag-tagged TRIM5α constructs at d1 using calcium phosphate precipitation and JetPEI (PolyPlus), respectively. Cells were lysed at d4 in 500 µl of IP lysis buffer and immunoprecipitation was performed as described above with anti-Flag antibody. The bound proteins Flag, TRIM5α, and SUMO1 were detected by Western blotting. All blots were acquired using the Chemidoc XRS+ imaging system and band quantifications were performed using the Image Lab^TM^ software.

### In vitro SUMO assay

Human TRIM5α recombinant protein was produced in *Escherichia coli* as a hexa-His fusion protein and purified by Ni-affinity chromatography (NIH AIDS Reagent Program). In vitro SUMO assay was performed using a SUMOylation kit by Enzo according to manufacturer’s protocol. Reactions containing SUMO1, E1, E2 (Ubc9) 200 nM of His-tagged HR-TRIM5α, and increasing amounts of RanBP2∆FG-GST (50, 100, or 200 nM) were incubated 60 min at 37 °C. Controls without ATP or E2 were included. Proteins were analyzed by western blotting using rabbit α-SUMO1 antibody provided by the Kit or mouse α-6×His antibody.

## Electronic supplementary material

Supplementary Information

## Data Availability

Authors confirm that all relevant data are available if requested.
