# Supplementary material for: RanBP2 regulates the anti-retroviral activity of TRIM5α by SUMOylation at a predicted phosphorylated SUMOylation motif
Source: Commun Biol. 2018 Nov 15;1:193. doi: 10.1038/s42003-018-0198-0 (PMC6237768; doi:10.1038/s42003-018-0198-0)
Supplement: Supplementary file 1 — Supplementary Information [file 42003_2018_198_MOESM1_ESM.pdf]

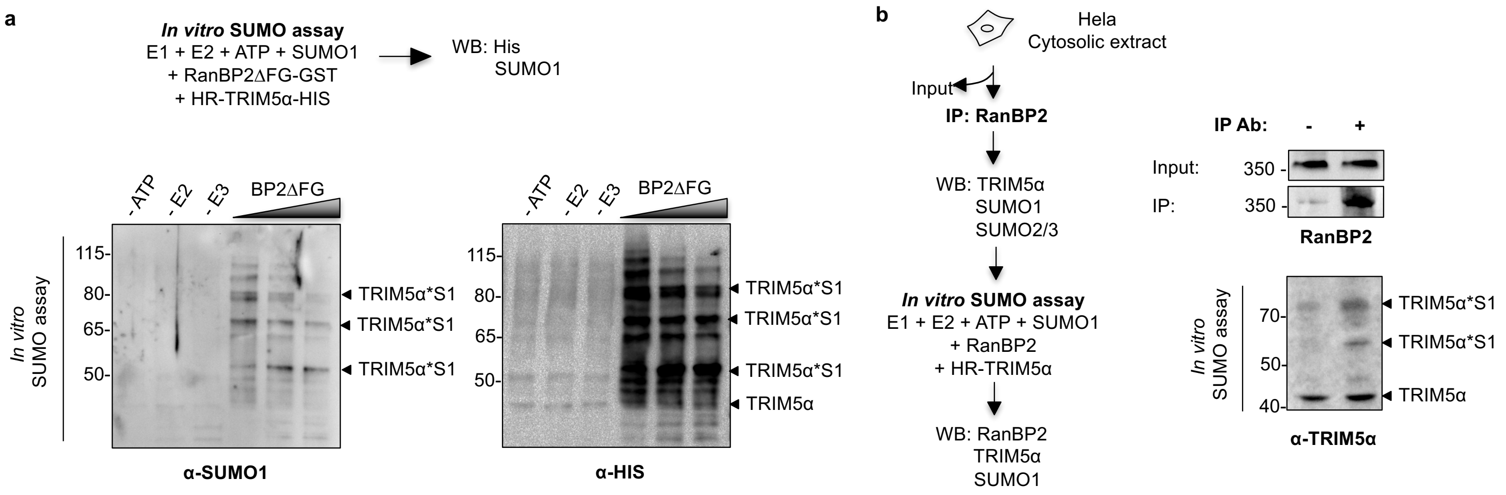

Supplementary Figure 1.
a, RanBP2 SUMOylates TRIM5α *in vitro*. *In vitro* SUMO assay was performed by combining His-tagged HR-TRIM5a (40 kDa), and the RanBP2 domain previously identified to contain E3 SUMO ligase activity of RanBP2 (RanBP2ΔFG, Pichler et al., 2002). Following *in vitro* SUMO assay, multiple SUMO modified TRIM5a bands, that appeared at *ca*. 15 kDa increments were visible in both SUMO1 and His blots, demonstrating that RanBP2ΔFG is sufficient to mediate TRIM5a SUMOylation *in vitro*.
b, Full length RanBP2 stimulates SUMOylation of recombinant TRIM5a *in vitro*. Human recombinant TRIM5α (HR-TRIM5α, 40 kDa) produced in E-*coli* was incubated with full-length human RanBP2 isolated from Hela cytosol. *In vitro* SUMOylation, performed in a minimal reaction assay containing RanBP2, TRIM5α, E1, E2, SUMO1 and ATP for 30 min at 30°C, generated two high molecular weight bands above the *ca.* 40 kDa unmodified band, at apparent mobilities of *ca.* 55 kDa and 70 kDa, indicating SUMO modification. Original blots are provided in Supplementary Figure 6).

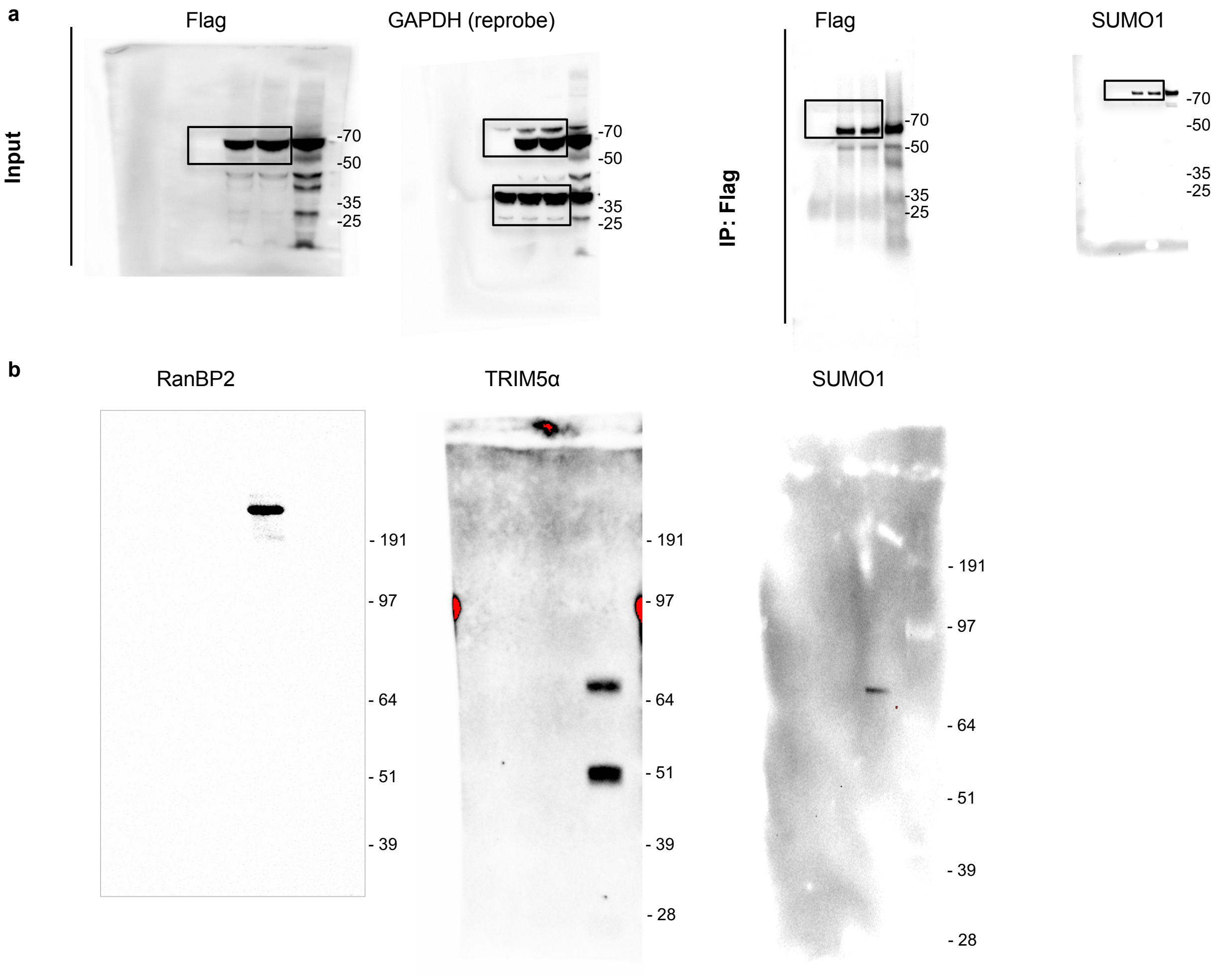

Supplementary Figure 2.
a, Original Western blots for Fig. 1a. **b,** Original Western blots for Fig. 1d.

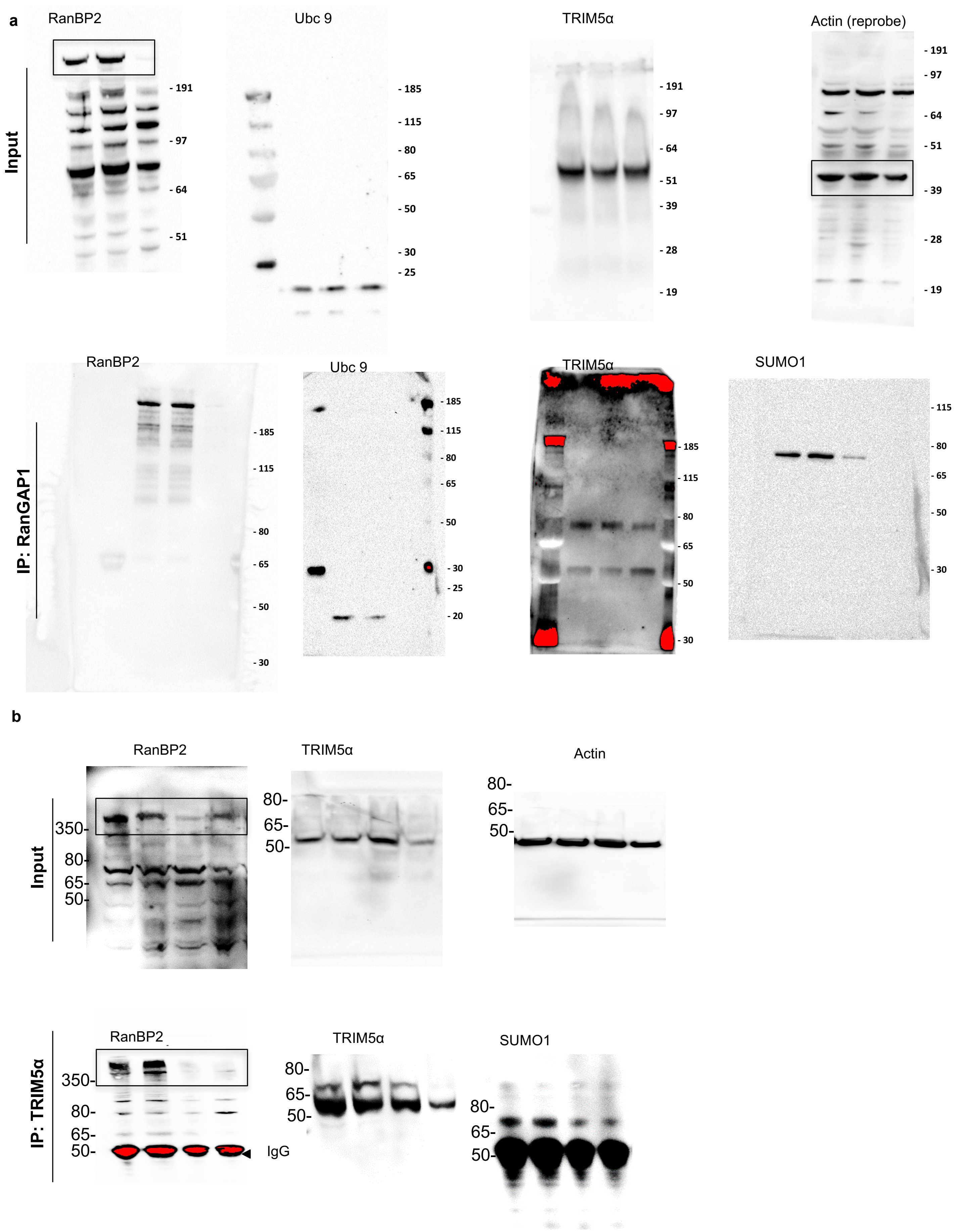

Supplementary Figure 3.
a, Original Western blots for Fig. 2a. **b,** Original Western blots for Fig. 2b.

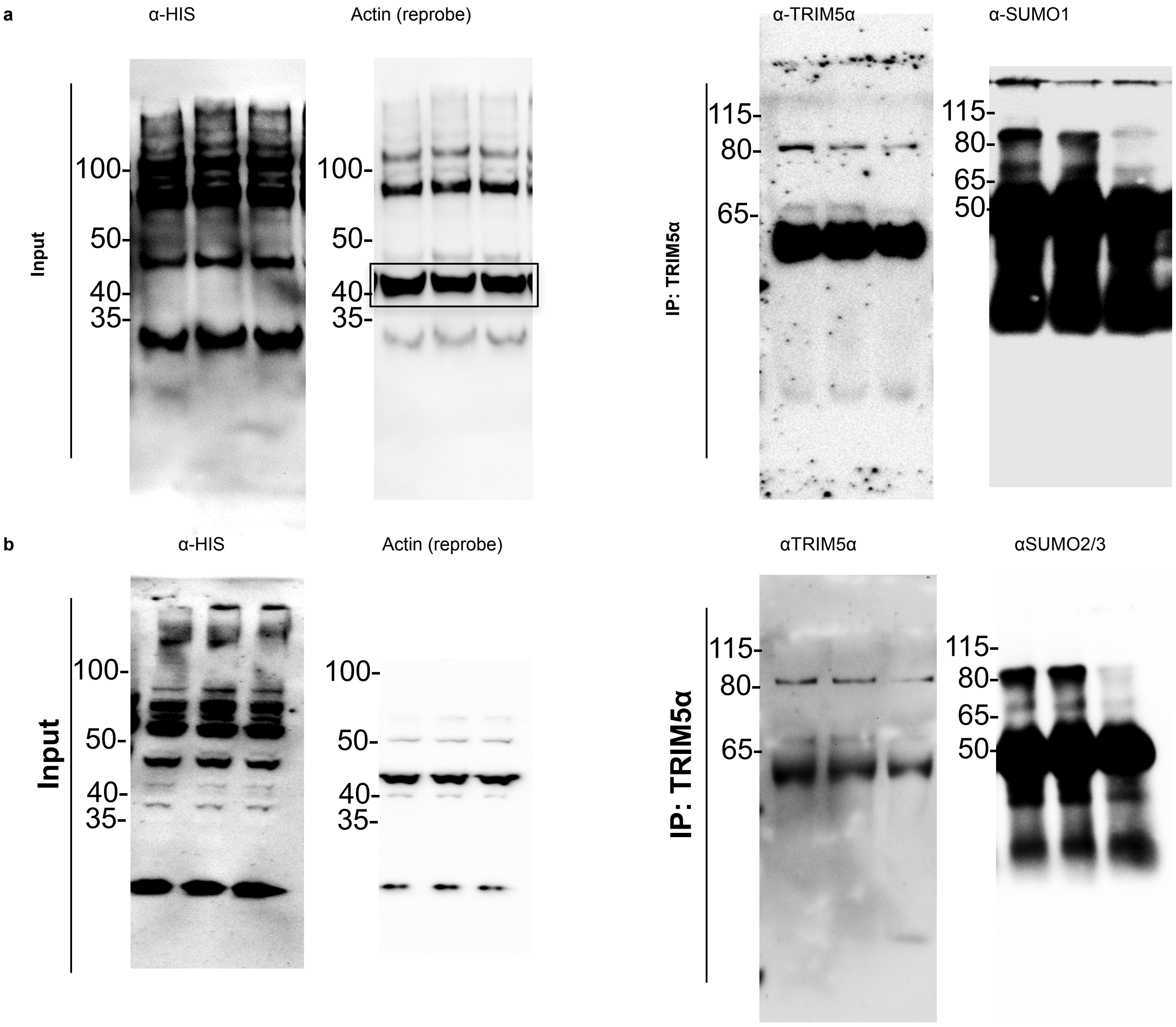

Supplementary Figure 4.

a, Original Western blots for Fig. 3d. **b**, Original Western blots for Fig. 3e.

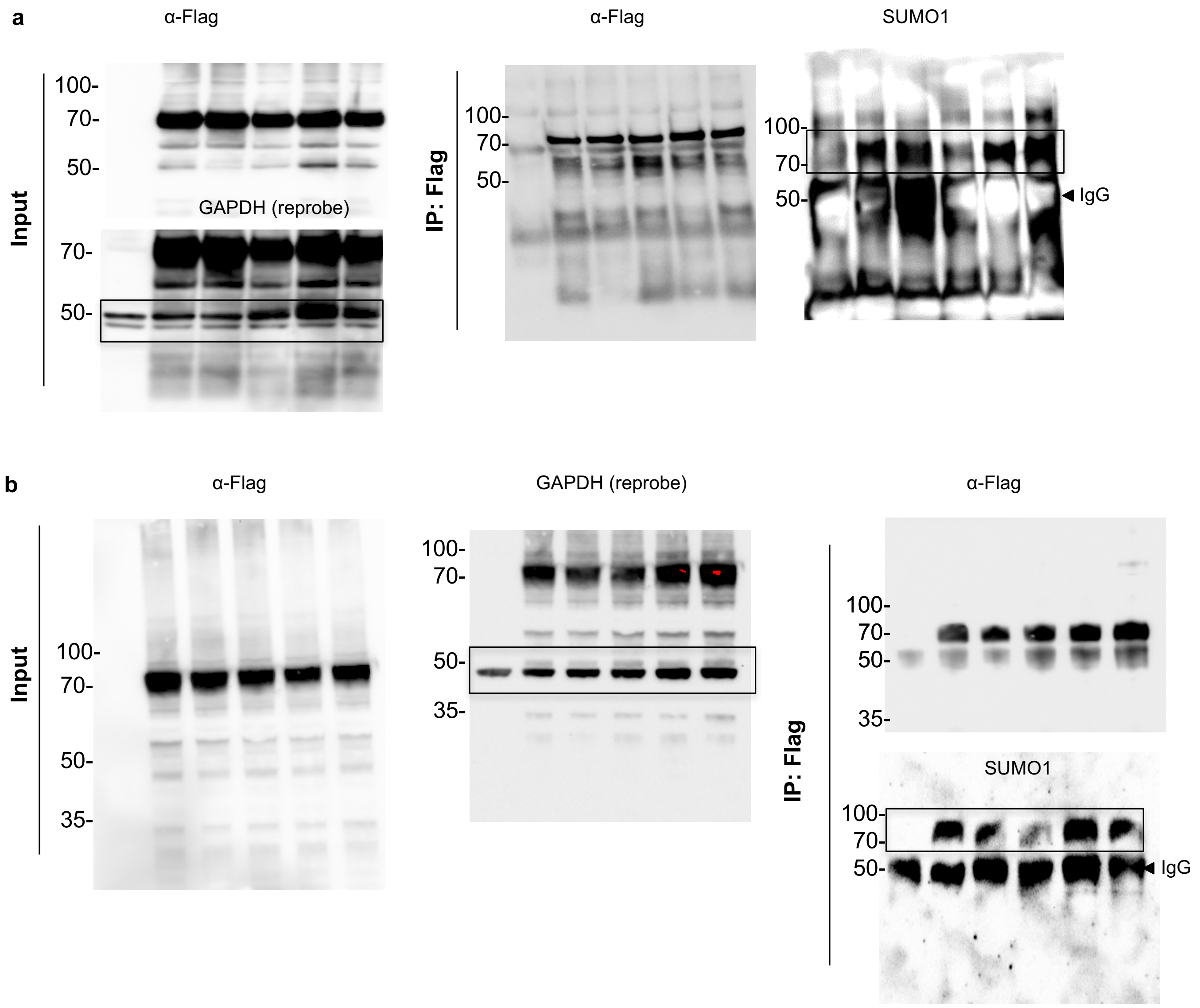

Supplementary Figure 5.
a, Original Western blots for Fig. 6b. **b**, Original Western blots for Fig. 6c.

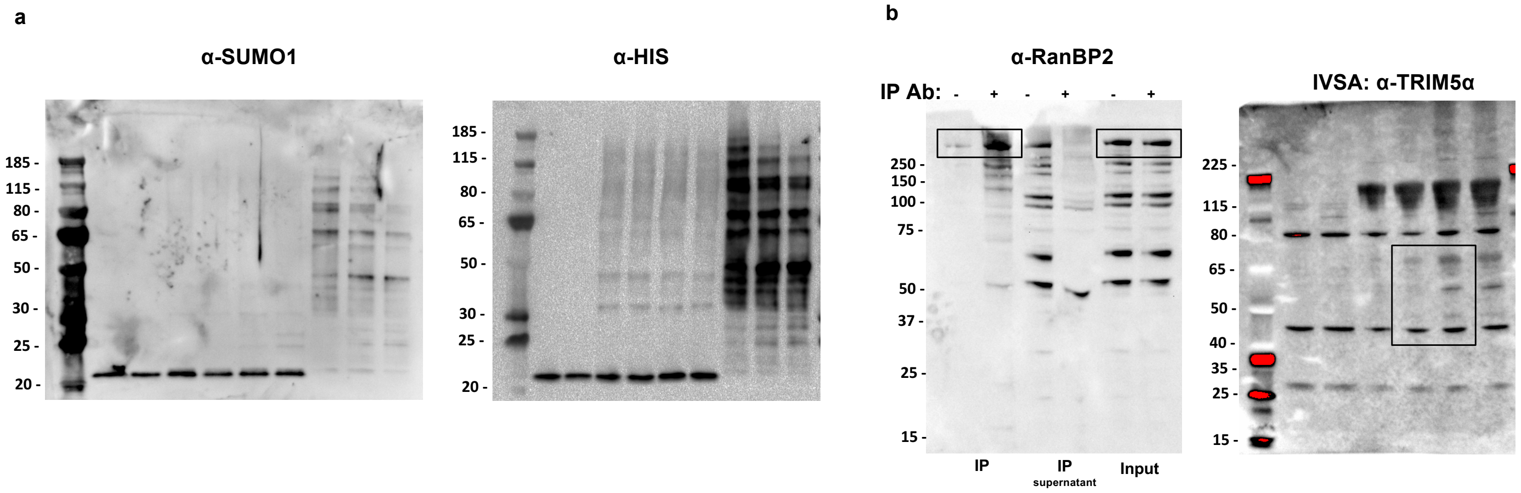

Supplementary Figure 6.

a, Original Western blots for Supplementary Figure 1a. **b**, Original Western blots for Supplementary Figure 1b.

Supplementary References:

Pichler, A., Gast, A., Seeler, J. S., Dejean, A. & Melchior, F. The nucleoporin RanBP2 has SUMO1 E3 ligase activity. *Cell* **108**, 109-120 (2002).
